# Environmental and occupational respiratory diseases – 1045. Skin prick tests and allergen-specific IgE tests for fungus in patients with chronic lower respiratory symptoms

**DOI:** 10.1186/1939-4551-6-S1-P44

**Published:** 2013-04-23

**Authors:** Jae Woo Jung, Jae Chol Choi, Jong Wook Shin, Jae Yeol Kim, In Won Park, Byoung Whui Choi

**Affiliations:** 1 Internal Medicine, Chung-Ang University Hospital, Seoul, South Korea

## Background

Skin prick tests (SPTs) has relatively a good correlation with allergen-specific IgE against house dust mites (HDM) and pollens, whereas their correlation to fungi remains low. We aimed to investigate the correlation between results of serum specific IgE and SPTs for HDM or fungi in patients with chronic lower respiratory symptoms. The clinical difference between serum fungus or HDM-specific IgE-positive and –negative groups was also examined.

## Methods

A total of 89 patients underwent both SPT and serum specific IgE test to D. farinae, D. pteronyssiuns and 5 fungi (*Penicillium notatum*, *Cladosporium*, *Aspergillus fumigatus*, *and Alternaria*,*Fusarium spp.*) with chronic lower respiratory symptoms were included in this study.

## Results

SPT and serum specific IgE tests for HDM were positive in 20.2% and 38.2% and for fungi 5.6% and 41.6%. The k statistic for the agreement between SPT and serum specific IgE test about HDM was relatively high (0.425, p<0.001) compared with fungi (k=0.102, P=NS). In patients with negative SPT to HDM, total IgE (741.15 vs. 84.33KU/L) was higher and FEV1 % predicted (76.95% vs. 90.33%) and PC20 (2.57 vs. 1.32mg/ml) were lower in the serum HDM-specific IgE positive group than in negative group (P<0.05). In patients with negative SPT to fungi, total IgE (480.32 vs. 119.67KU/L) was higher in the serum fungi-specific IgE positive group than in negative group (P<0.05).

## Conclusions

The rate of successful detection of HDM and fungi using SPT was low compared to serum specific IgE. Thus, serum specific IgE might need to detect HDM and fungus allergies in patients with chronic lower respiratory symptoms.

